# Motion guided segmentation of the right ventricle for 3D cine DENSE MRI

**DOI:** 10.1186/1532-429X-15-S1-P82

**Published:** 2013-01-30

**Authors:** Andrew D Gilliam, Frederick H Epstein

**Affiliations:** 1 A.D. Gilliam Consulting, Providence, RI, USA; 2 Biomedical Engineering, University of Virginia, Charlottesville, VA, USA; 3 Radiology, University of Virginia, Charlottesville, VA, USA

## Background

Cine displacement encoding with stimulated echoes (DENSE) provides quantitative imaging of tissue motion with high spatial resolution. Recent results have demonstrated that 3D cine DENSE provides resolution sufficient to quantify the mechanics of the right ventricle (RV) [1]. However, RV analysis currently requires manual anatomical delineation on all cine frames. This research extends motion guided segmentation methods [2,3] to the RV, requiring manual delineation of anatomy on only a single cine frame.

## Methods

Five healthy volunteers were imaged using a 3D cine DENSE CMR protocol on a 1.5T MR system (Avanto, Siemens). Imaging was performed with informed consent and in accordance with protocols approved by our Institutional Review Board.

DENSE observes tissue displacement at fixed voxels through which the underlying tissue moves. Each phase observation is proportional to a displacement indicating the initial location of underlying tissue when DENSE encoding occurred. Large phase values are wrapped to the intrinsic phase range of [-π,π]. Voxels empty of tissue contain unpredictable phase information.

Our solution propagated user-defined end-systolic RV anatomy through the multi-phase image series, using displacement guidance provided by DENSE observations. Voxels crossed by the end-systolic RV surface were identified, phase wrapping artifacts were corrected via a quality guided path following algorithm, and the RV was projected to its initial configuration via 3D radial basis function (RBF) interpolation. On each frame, we predicted the current RV configuration according to a linear motion model, unwrapped DENSE observations consistent with neighboring predictions, and estimated the true RV configuration from unwrapped observations via 3D RBF interpolation.

## Results

**Figure 1:**
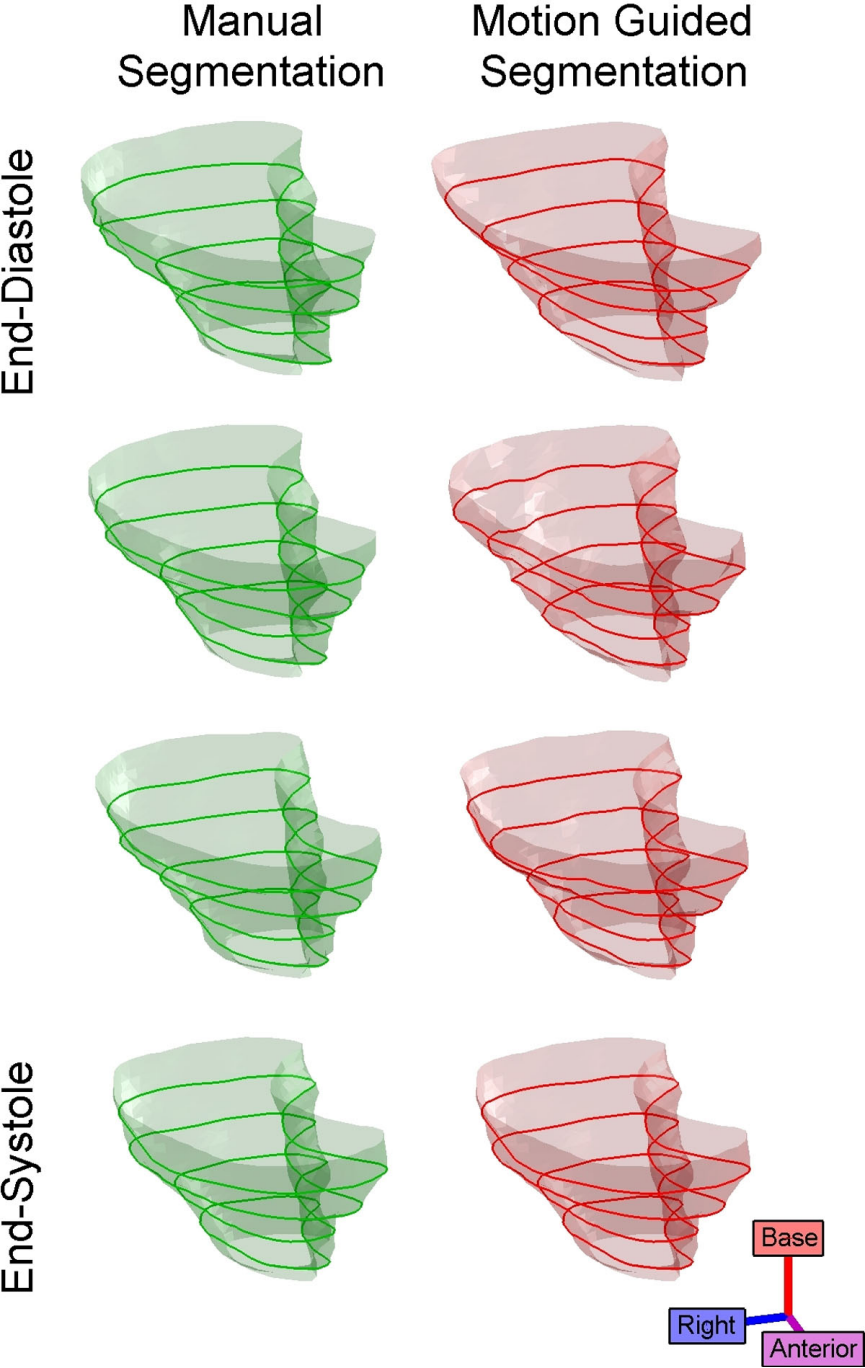
Right ventricular anatomy in a healthy human volunteer; (left column) manual segmentation, (right column) motion guided segmentation.

Anatomy was manually delineated on all frames of all datasets for comparison. Fig. 1 illustrates a typical motion guided segmentation result compared to manual. Automatically guided segmentations achieved a root mean square distance of 1.73 mm to manual segmentations, with 92% of points on the guided segmentation surfaces within one pixel of manual segmentation surfaces.

## Conclusions

This research presents the first motion guided segmentation algorithm specific to the RV for 3D cine DENSE CMR. This method promises to substantially reduce the time required for strain analysis of the RV. Guided segmentation results agreed well with manual RV delineation. Ongoing work seeks to further refine the automation process, perhaps eliminating the need for user-defined anatomy entirely.

## Funding

Supported in part by NIH R01 EB 001763, Siemens, and AHA 12GRNT12050301.
